# An approach to autologous stem cell mobilization: trying to define good mobilizers

**DOI:** 10.1016/j.htct.2024.04.126

**Published:** 2024-09-07

**Authors:** Sara Montolio Chiva, Paula Gomez Fernandez, Antonio Manuel Gutiérrez Garcia, Maria del Carmen Ballester Ruiz, Antonia Sampol Mayol, Albert Perez Montaña

**Affiliations:** Hospital Universitari Son Espases, IdISBa, Palma de Mallorca, Spain

**Keywords:** Autologous stem cell transplantation, G-CSF, Mobilization, CD34+ cells, Good mobilizers

## Abstract

**Background and objectives:**

Stem cell mobilization is a well-known procedure to harvest hematopoietic stem cells for autologous stem cell transplantation in certain hematologic diseases. Numerous studies have been conducted to identify risk factors for poor mobilization but there are no studies that identify good mobilizers. In our hospital, we decided to explore good mobilizers, defining them as those with ≥40 CD34^+^ cells/μL on Day +4 in order to start early apheresis.

**Material and methods:**

A descriptive retrospective study was performed at Hospital Universitari Son Espases. A total of 198 patients mobilized with doses of around 10 µg/kg of granulocyte colony-stimulating factor (G-CSF) every 12 h were analyzed for autologous collection between January 2015 and September 2022. Fifty patients who had ≥40 CD34^+^ cells/μL on Day +4 started early apheresis; the rest continued mobilization as planned. Success was defined as obtaining over 2.5 × 10^6^ CD34^+^ cells/kg in a single apheresis.

**Results:**

The necessary number of CD34^+^ cells/kg to perform an autologous stem cell transplantation was reached in a single apheresis session in 62 % of patients with ≥40 CD34^+^ cells/μL in peripheral blood. A cutoff of 102 CD34^+^ cells/μL on Day +4 was shown to have the best success rate (94 %)**.** In an analysis of success, age, previously failed mobilization and having one or more adverse factors for bad mobilization were statistically significant.

**Conclusion:**

Patients considered as good mobilizers were matched with our factors of poor mobilization, revealing that most patients (79 %) had none or only one risk factor for poor mobilization. Apheresis on Day +4 in good mobilizers was shown to be an effective alternative to reduce mobilization duration and decrease the amount of granulocyte-colony stimulating factor administered.

## Introduction

Autologous stem cell transplantation (ASCT) consists of the administration of intensive chemotherapy followed by the infusion of the patientʼs own hematopoietic stem cells (HSC) with a potentially curative intention in certain hematologic neoplasms^1^ and also in some non-hematologic diseases. These cells can be obtained from bone marrow, umbilical cord blood, or peripheral blood (PB), the latter being the most widely used nowadays.^2^^,^^3^ Under normal conditions, the number of HSC in peripheral blood is lower than in the bone marrow. In most centers, a minimum of two million CD34^+^ cells/kg is required to perform an ASCT.^4^, ^5^, ^6^, ^7^ By administering granulocyte colony-stimulating factor (G-CSF) alone or in combination with chemotherapy, HSC move out of the marrow compartment into the PB, a process known as mobilization.^2^^,^^3^^,^^6^^,^^8^

Generally, the mobilization process lasts at least five days when G-CSF is used alone.^9^^,^^10^ The standard dose of G-CSF used is between 5 and 10 µg/kg every 12 h.^11^ On the 5th day, apheresis is usually performed using automatic separators to harvest HSC.^12^

The collection of HSC is usually guided by the CD34^+^ cell count in PB on Day +4 of mobilization. A correlation has been observed between the number of circulating CD34^+^ cells in PB during mobilization and the number of CD34^+^ cells obtained through the apheresis procedure.^4^, ^5^, ^6^^,^^13^, ^14^, ^15^

Multiple reviews have tried to identify prognostic factors for poor mobilization.^6^^,^^13^^,^^16^, ^17^, ^18^, ^19^, ^20^ In our center, the consensus of Catalan-Balearic clinical experts is used as a guide to identify the factors that predict whether a patient has a greater or lesser risk of being poorly mobilized.^5^^,^^21^ These predictors of inadequate mobilization are being over 65 years old, cytopenia (thrombocytopenia: platelets <100 × 10^9^/L; neutropenia: neutrophils <1 × 10^9^/L), bone marrow infiltration at the time of diagnosis in the case of lymphomas, diagnosis of mantle cell lymphoma, two or more lines of previous chemotherapy, extensive radiotherapy,^22^ previous treatment with lenalidomide, fludarabine, ibritumomab or thioxetane,^23^ or previous mobilization failure.^24^

However, the management of patients who are considered very good mobilizers has not been explored as much as poor mobilizers. There is no consensus to define good mobilizers in accordance with CD34^+^ cell counts. In our center, we considered the possibility of bringing HSC apheresis forward to Day +4 of mobilization in patients with high CD34^+^ cell counts. The cutoff point was arbitrarily set at ≥40 CD34^+^ cells/μL in PB to start the patient's apheresis.

The objective of this study was to evaluate the efficacy of performing early HSC apheresis in good mobilizers. A secondary objective was to define an optimal cutoff point of CD34^+^ cells to decide when to start apheresis in good mobilizers. At the same time, to see whether this change could reduce the number of days of mobilization and therefore the total dose of G-CSF administered.

## Study design and methods

### Patient selection

This retrospective descriptive study was carried out at Hospital Universitari Son Espases (HUSE). To avoid selection bias, patients were selected from the databases of the Apheresis and Cellular Therapy Sector of the Hematology Service. Data from 198 mobilizations with G-CSF alone between January 2015 and September 2022 were analyzed. These 198 mobilizations were performed in 183 patients, with 15 patients having two mobilization procedures due to previous mobilization failure or a late relapse that required a second transplant. Patients with ≥40 CD34^+^ cells on Day +4 were selected. No exclusion criteria were applied. Clinical-biological variables (age, gender, and diagnosis) were analyzed, along with the presence of predictive factors for poor mobilization, G-CSF dose administered, baseline CD34^+^ cell count obtained on Day +4, the number of blood volumes processed during apheresis, total CD34^+^ cells obtained in the first apheresis, and the number of apheresis procedures necessary to reach the established minimum number of cells to ASCT. The study was approved by the institutional ethics committee (IB 5183/23 PI).

### Mobilization and apheresis procedures

All patients had the same mobilization protocol. G-CSF subcutaneous injections were administered at 10 µg/kg/12 h.

All patients underwent a blood test on Day +4. Those with <10 CD34^+^ cells/μL received plerixafor in combination with G-CSF; those who had between 10 and 40 CD34^+^ cells/μL continued with G-CSF, starting apheresis the day after. Those who obtained ≥40 CD34^+^ cells/μL started the process of apheresis in advance on the fourth day and were considered to be good mobilizers. As mentioned above, a cutoff point of ≥40 CD34^+^ cells/μL was selected arbitrarily.

To collect CD34^+^ cells from the PB, an Optia Spectra® (Terumo BCT) or Amicus® (Fresenius Kabi) automated cell separators were used with the Optia Spectra® being used the most. Apheresis duration was from four to five hours; with a mean of two to three blood volumes being processed. A maximum number of three apheresis procedures was established.

Since 2015, a minimum cellularity of 2.5 × 10^6^ CD34^+^ cells/kg has been established in our hospital as the objective to proceed to the ASCT.

Despite this, patients who can collect between 2 and 2.5 × 10^6^ CD34^+^ cells/kg are usually accepted and are not forced to undergo another apheresis procedure.

Generally, in young patients with multiple myeloma (MM) – especially those with high cytogenetic risk – the plan is to collect a minimum of 5 × 10^6^ CD34^+^ cells/kg to be able to perform a tandem transplant.

### Statistical methods

Variables following binomial distributions (i.e.: sex, success rate), are expressed as frequencies and percentages. Comparisons between qualitative variables were conducted using the Fisher exact or Chi-square tests. Comparisons between quantitative and qualitative variables were performed through non-parametric tests (Mann–Whitney U or Kruskal–Wallis U tests). Binary logistic regression was used to analyze factors related to apheresis success.

## Results

### Characteristics of patients

A total of 198 mobilizations were performed between January 2015 and September 2022. Of all the patients mobilized in this period, 50 had ≥40 CD34^+^ cells/μL on Day +4.

These patients were considered good mobilizers. Patients’ characteristics are listed in Table 1. Briefly, of the 50 patients analyzed, 28 were men (56 %) with a mean age of 53 years (range: 17–74) and only five were over 65 years old (10 %).

**Table 1:** Clinical characteristics of good-mobilizer patients.

**Characteristic**	
Median age (range)	53 (17–74)
Sex (male/female)	28 (56 %) / 22 (44 %)
Diagnosis:	
Plasma cell disorders^a^	30 (60 %)
Lymphoma	18 (36 %)
Other	2 (4 %)
Risk factors for bad mobilizers:	
0	19 (38 %)
1	22 (44 %)
2–3	9 (18 %)

a Plasma Cell Disorders include multiple myeloma, amyloidosis, solitary plasmacytoma, and POEMS syndrome.

Concerning the hematologic or non-hematologic diseases that had led to this procedure, 22 patients (44 %) had a diagnosis of MM; 13 (26 %) had non-Hodgkin lymphoma (NHL) (11 of B-cell lineage and two of T-cell lineage); five (10 %) Hodgkin lymphoma (HL); six (12 %) amyloidosis; one (2 %) solitary plasmacytoma; one (2 %) POEMS syndrome; one (2 %) Behçet's syndrome; and one (2 %) a testicular tumor.

### Mobilization and apheresis

Following the Catalan-Balearic consensus,^5^ 19 (38 %) patients did not present any prognostic factors for poor mobilization, 22 (44 %) patients had one, and nine (18 %) patients had two or three.

The median dose of G-CSF administered was 9.1 µg/kg/12 h (range: 4.5–11.4 µg/kg). All patients underwent a blood test on Day +4 of mobilization. The median number of CD34^+^ cells obtained in this cohort was 77.5 (range: 41–477). To collect the CD34^+^ cells, 40 (80 %) patients were connected exclusively to the Optia Spectra® cell separator to perform the apheresis while the Amicus® was used in eight (16 %) patients and only two (4 %) used both (one apheresis session with each).

The median number of processed blood volumes in the first apheresis was two (range: 1.42–3.1). The median number of CD34^+^ cells obtained in the first apheresis performed on Day +4 of mobilization was 3.2 × 10^6^ cells/kg (range: 0.4–27.5). The median of total CD34^+^ cells obtained with apheresis was 5.60 × 10^6^ cells/kg (range: 1.19–27.49). As explained above, in our center the objective is to obtain a minimum of 2.5 × 10^6^ CD34^+^ cells/kg to carry out an ASCT. The definition of success was established as obtaining that amount by apheresis on Day +4.

Of the 50 cases, 31 patients (62 %) achieved ≥2.5 × 10^6^ CD34^+^ cells/kg in the first apheresis. The 31 patients who only required one apheresis had the following diagnoses: 12 MM (39%), nine NHL (29 %), six HL (20 %), two amyloidosis (6 %), one POEMS Syndrome (3 %), and one testicular tumor (3 %). Twenty-six (52 %) of the 50 patients had one apheresis performed, 16 (32 %) had two performed, and two (4 %) had three; in six patients (12 %), two apheresis were performed despite having obtained more than 2.55 × 10^6^ of CD34^+^ cells/kg in the first apheresis. These six patients had a diagnosis of MM (most had high cytogenetic risk and were young); the objective of the second collection was to harvest > 5 × 10^6^ CD34^+^ cells/kg because of the possibility of performing a tandem ASCT or the future need to perform a second transplant. Interestingly, if the objective was reduced to 2 × 10^6^ CD34^+^ cells/kg – which is the minimum to perform a transplant in many centers – the number of patients reaching success increased to 78 %. The results regarding apheresis data are given in Table 2.

**Table 2:** Mobilization data and apheresis results.

**Characteristic**	
Median dose of G-CSF - µg/kg (range)	9.1 (4.5–11.4)
Automated cell separator – apheresis sessions (%)	
Optia Spectra®	40 (80 %)
Amicus®	8 (16 %)
Both	2 (4 %)
CD34^+^ peak x10⁶ - median (range)	77.5 (41–477)
CD34^+^ cells/kg 1st apheresis – median (range)	3.2 (0.4–27.5)
≥2.5 × 10^6^ CD34^+^ cells/kg 1st apheresis – patients (%)	31 (62)
≥2 × 10^6^ CD34^+^ cells/kg 1st apheresis – patients (%)	39 (78)

### Optimal cutoff for mobilization

**Figure 1:**
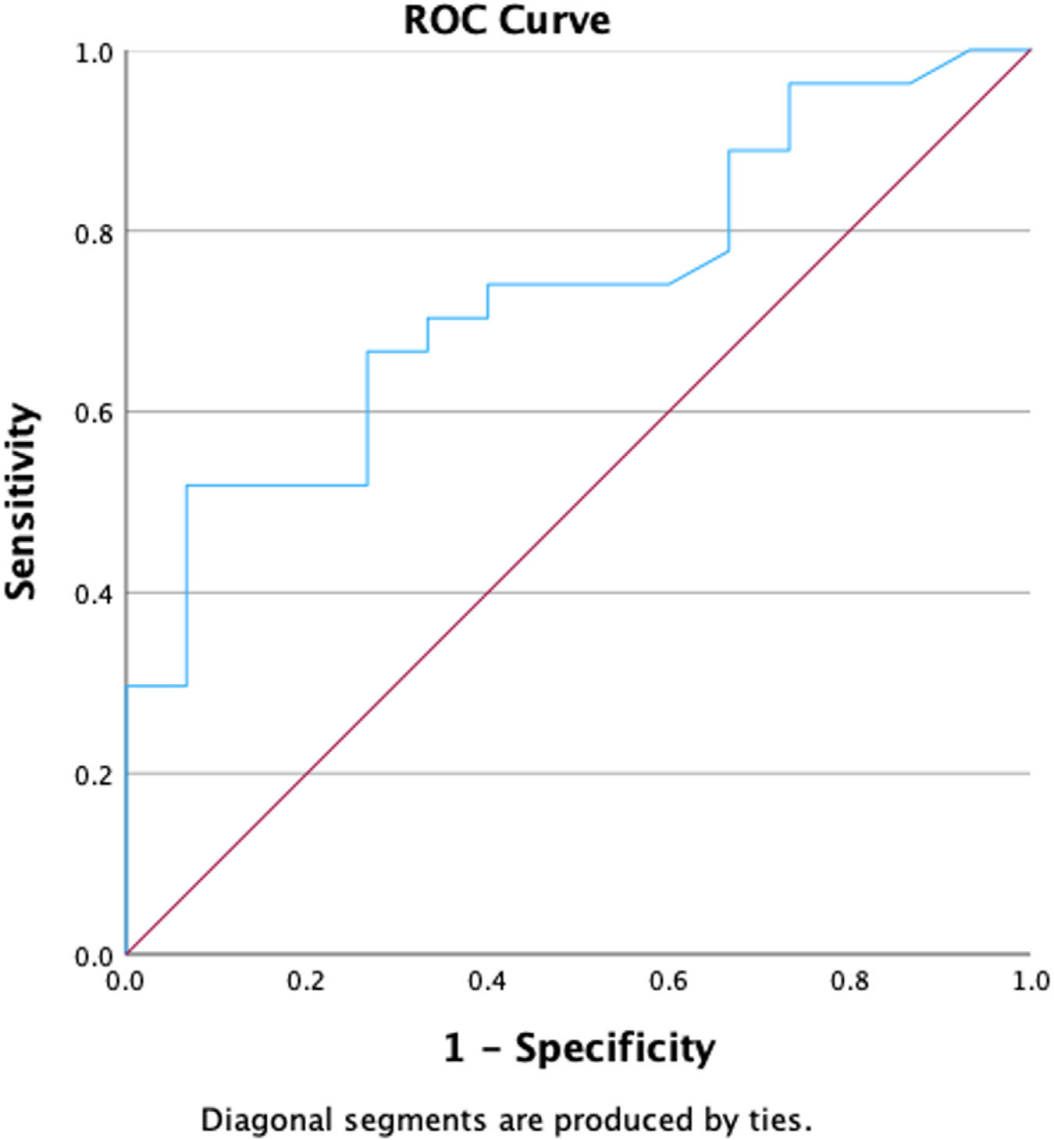
Receiver operating characteristic (ROC) curve for calculating the optimal cutoff for obtaining successful apheresis.

The relationship between the number of CD34^+^ cells/µL detected in PB on Day +4 of mobilization and the option for obtaining ≥2.5 × 10^6^ CD34^+^ cells/kg in the first apheresis were analyzed. Receiver operating characteristic (ROC) curves, used to calculate the best cutoff to start apheresis, gave an optimal amount of 102 CD34^+^ cells/μL of PB on Day +4 (area under curve [AUC]: 0.73; *P*-value = 0.013 - Figure 1). With this cutoff, a success rate of 94 % was obtained on the first day of apheresis. However, as this optimal cutoff is not always easily obtainable, different ranges of CD34^+^ cells/µL were also tested, starting from the lowest value (40 CD34^+^ cells/µL) and increasing 10 by 10 CD34^+^ cells/µL with the percentage of patients achieving success being calculated. These results are shown in Table 3. The success rate increased from 62 % when the cutoff was 40 CD34^+^ cells/µL to 94 % when the cutoff was 102 CD34^+^ cells/µL. No significant differences in success were found when the cutoff was increased beyond the 102 CD34^+^ cells/µL mark.

**Table 3:** Relationship between cutoff of CD34^+^ (× 10⁶ cells/kg) on Day +4 and mobilization success rate of the first apheresis session.

Cutoff CD34+ cells/kg on Day +4	Success rate (2.5 × 10^6^ CD34^+^ cells/kg on 1st day) (%)
40	62 %
50	64 %
60	68 %
70	73 %
80	79 %
90	75 %
102	94 %

### Analysis of factors associated with apheresis success

Both univariate and multivariate analyses were performed to analyze the factors associated with apheresis success, including age, diagnosis, number of prognostic factors of poor mobilization (0 versus 1–3), the automated cell separator used, and baseline CD34^+^ cell count in PB. As shown in Table 4, only baseline CD34^+^ cell counts on Day +4 (*P*-value = 0.002) and lymphoma diagnosis (plasma cell disorders versus lymphoma - *P*-value = 0.041) were significantly associated with apheresis success. However, the presence or absence of risk factors for poor mobilization seemed to tend towards statistical significance (*P*-value = 0.074).

**Table 4:** Analysis of clinical factors associated with mobilization success rate.

Univariate analysis	Success	*P*-value
Age -%		0.56
≤ 60 years	64	
> 60 years	40	
Diagnosis -%		0.041
Plasma Cell Disorder	50	
Lymphoma	80	
Risk factors for poor mobilization – n (%)		0.074
0	15 (79)	
1–3	16 (52)	
Automated cell separators – n (%)		1
Optia Spectra®	25 (62)	
Amicus®	6 (67)	
Blood CD34^+^ counts (cells/µL) – n (%)		0.002
40–101	16 (47)	
≥ 102	15 (94)	
**Multivariate analysis**	RR (95 %CI)	*P*-value
Plasma Cell Disorder	3 (0.7–13)	0.15
1–3 risk factors for poor mobilization	2.4 (0.5–10.9)	0.25
Baseline CD34^+^ cells/kg <102 cell/μL	10.4 (1.1–93.8)	0.037

RR: risk ratio; 95 %CI: 95 % confidence interval.

Of the 50 patients considered good mobilizers, 31 patients achieved success. Analyzing only these patients, 15 of them did not present risk factors for poor mobilization while 16 patients presented 1–3 risk factors. These data imply that out of all the good mobilizers with no risk factors, 15/19 (79 %) achieved success; whereas of those with 1–3 risk factors, only 16/31 (52 %) achieved success.

Further, when including all these three variables in a multivariate analysis, only the peak of the CD34^+^ cell count on Day +4 < 102 cells/μL was found to influence unsuccessful apheresis (risk ratio: 10.4; 95 % confidence interval: 1.1–93.8; *P*-value = 0.037).

Additionally, we decided to compare patients considered good mobilizers with the rest (normal and bad mobilizers). Age, diagnosis, risk factors for being a bad mobilizer, and number of risk factors were analyzed (Table 5). The following were obtained as significant factors: age, previous mobilization failure, and the presence of risk factors for being bad mobilizers.

**Table 5:** Comparison of good and normal-bad mobilizers on Day +4.

	Good mobilizer (*n* = 50)	Normal-bad mobilizer (*n* = 148)	*P*-value
Median age - years (range)	53 (17–74)	58 (1–71)	0.11
Diagnosis - n (%):			0.45
- Plasma Cell Disorder - Lymphoma - Solid tumors - Autoimmune diseases - Other	30 (60)	91 (61)	
	18 (36)	43 (29)	
	1 (2)	12 (8)	
	1 (2)	1 (1)	
	0 (0)	1 (1)	
Risk factor for bad mobilization - n (%):			
- Age - Lenalidomide - BM infiltration - Radiotherapy - Mantle cell lymphoma - > 2 previous treatment lines - Previous mobilization failure	4 (8)	33 (22)	0.034
	16 (32)	64 (43)	0.18
	3 (6)	14 (9)	0.64
	4 (8)	20 (13)	0.45
	4 (8)	8 (5)	0.75
	4 (8)	21 (14)	0.33
	0 (0)	18 (12)	0.021
Risk factors for poor mobilization – n (%):			<0.001
- 0 - 1 or more	19 (38)	21 (14)	
	31 (62)	127 (86)	

In relation to age, 8 % of good mobilizers were older than 65, whereas this was 22 % in the case of normal and bad mobilizers (*P*-value = 0.034). Regarding mobilization failure, good mobilizers had no previous history of failure (0 %); on the other hand, in normal and bad mobilizers, previous failure was present in 12 % (*P*-value = 0.021). No significant results were obtained for the other variables.

## Discussion

HSC mobilization is a highly standardized procedure with few differences in the way of proceeding in apheresis services. Management of poor mobilizers is well defined, both in its definition and in the associated use of both plerixafor and G-CSF to increase the release of HSC into the PB ^4^^,^^6^^,^^11^.

Poor mobilization and its predictive factors has been highly studied with different results.^5^^,^^16^, ^17^, ^18^, ^19^ Nevertheless, there is no international consensus as to which of these factors should be used in the clinical practice or any prognostic score to accurately define the risk of failure.

In contrast, there are no guidelines or protocols focused on defining or managing patients with a high capacity to release CD34^+^ cells, who can be called good mobilizers.

For this reason, this study explores the benefits of performing an early apheresis on Day +4 of mobilization in patients with particularly high CD34^+^ cell counts. After reviewing the literature, we did not find any articles that studied the use of early apheresis to compare with our results. Due to the absence of data concerning this topic we arbitrarily decided to take 40 CD34^+^cells/µL as the cutoff to define good mobilizers and thereby bring apheresis forward to Day +4.

As mentioned above, in terms of predictive factors for poor mobilization, we followed the committee of experts of the Catalan-Balearic Hematology Society to which we belong.^5^ In this study, most of our patients considered good mobilizers were found to barely have any risk factors for poor mobilization: 82 % had none or only one while 18 % had two or three.

In addition, it is worth mentioning that 16 patients out of the 50 considered as good mobilizers had a history of previous treatment with lenalidomide. All of them had a diagnosis of MM. Since 2017, all our MM patients have received treatment with the VRD scheme (bortezomib + lenalidomide + dexamethasone) as induction therapy. In these patients, mobilization is performed after the third cycle to achieve a good enough response and avoid prolonged exposure to lenalidomide.

The impact of prior lenalidomide exposure has been studied in many articles with contradictory results.^25^, ^26^, ^27^ The adverse effect of lenalidomide on the ability of HSC harvesting is well known, especially after prolonged exposure. Cavallo et al.^28^ published an article analyzing 346 patients with newly diagnosed MM who had received four cycles of lenalidomide as induction therapy prior to mobilization. Their objective was to achieve 4 × 10^6^ CD34^+^ cells/kg. The median number of apheresis procedures was three with 91 % of patients obtaining enough HSC to perform an ASCT.

Moreover, Johnsrud et al.^29^ measured the impact of previous lenalidomide use on mobilization outcomes. They stratified patients in three groups related to exposure to lenalidomide: no exposure, <6 cycles, and ≥6 cycles. No difference in cell yield was obtained between no exposure and <6 previous cycles but patients who received ≥6 cycles had a lower CD34^+^ cell yield.

According to the literature, it seems that a short induction therapy with lenalidomide is feasible to obtain a satisfactory yield of CD34^+^ cells, making this drug an exposure-dependent adverse factor.

There is no consensus regarding the adverse risk factors for poor mobilization but, as shown in the current study, those from the Catalan-Balearic group allowed us to discriminate a better group of mobilizers (0–1 risk factors) and a worse group (≥2 risk factors), important to define the good and also bad mobilization patients. More studies are needed to better define adverse risk factors in order to achieve a unanimous consensus.

To define good mobilizers, it seems reasonable to think that, independently from the adverse risk factors, patients with a high number of CD34^+^ cells/μL on Day +4 of mobilization would cope well with early apheresis, as demonstrated.

This study found that 62 % of patients above the arbitrarily chosen cutoff of 40 CD34^+^ cells/μL achieved success with only one apheresis session, but as mentioned previously, success increased to 78 % when the objective was reduced to 2 × 10^6^ CD34^+^ cells/kg. This seems a very interesting result because it enables the total time spent on the mobilization process to be reduced in two out of every three patients (three out of four in the case of the reduced objective).

According to this analysis, we showed that by choosing 102 CD34^+^ cells/μL as the cutoff, 94 % of our patients achieved success with just one apheresis on Day +4, making it an excellent discriminator of good mobilizers. However, the cutoff of 102 CD34^+^ cells/μL seems too high to be used in the daily clinical practice. More studies with a higher number of patients could reveal a lower result with a similar high success rate.

## Conclusion

In summary, steady state mobilization is a well-known process, and strategies to optimize patients classified as bad mobilizers with the use of plerixafor are well established. Even so, a major consensus to design a score that enables a better prediction of the patients that could benefit with pre-emptive use of plerixafor or other booster strategies is missing. Despite this, the criteria we use showed that there is a relationship between the absence of adverse risk factors and good mobilization, as 82 % of the patients considered good mobilizers had none or just one adverse risk factor.

Nonetheless, as shown in this study, there is still much to be done, in the field of the subgroup of patients that respond better than average. Due to the lack of supporting literature, we chose an arbitrary cutoff of 40 CD34^+^ cells/μL on Day +4 which turned out to be a good predictor of successful apheresis. However, more studies are needed to better define which patients are good mobilizers, when it is best to carry out an anticipated apheresis, and what is the best cutoff.

## Author contributions

P.G. and S.M. collected patient data and wrote the manuscript. A.P. designed the study, performed methodology, reviewed, and edited the manuscript. A.G conducted the statistical analysis and data interpretation. A.P. and C.B. contributed by visiting patients. A.S. provided infrastructure and patients.

## Funding

This research did not receive any specific grant from funding agencies in the public, commercial, or not-for-profit sectors.

## Conflicts of interest

The authors declare no conflict of interest.

## Data Availability

The datasets used and/or analyzed during the current study are available from the corresponding author on reasonable request.
